# 同基因造血干细胞移植治疗再生障碍性贫血11例疗效分析

**DOI:** 10.3760/cma.j.issn.0253-2727.2021.06.006

**Published:** 2021-06

**Authors:** 樱 张, 栋林 杨, 荣莉 张, 卫华 翟, 爱明 庞, 祎 何, 尔烈 姜, 四洲 冯, 明哲 韩

**Affiliations:** 中国医学科学院、北京协和医学院血液病医院（中国医学科学院血液学研究所），实验血液学国家重点实验室，国家血液系统疾病临床医学研究中心，天津 300020 State Key Laboratory of Experimental Hematology, National Clinical Research Center for Blood Diseases, Institute of Hematology & Blood Diseases Hospital, Chinese Academy of Medical Sciences & Peking Union Medical College, Tianjin 300020, China

**Keywords:** 同基因, 造血干细胞移植, 再生障碍性贫血, 植入失败, Syngeneic, Hematopoietic stem cell transplantation, Aplastic anemia, Graft failure

## Abstract

**目的:**

评价同基因造血干细胞移植（syn-HSCT）治疗再生障碍性贫血（AA）的疗效。

**方法:**

对11例接受syn-HSCT的AA患者进行回顾性分析。

**结果:**

全部11例AA患者中男4例，女7例，中位年龄为22（7～44）岁。全部11例患者移植后均获得造血重建，中位粒细胞植入时间为10（8～23）d，中位血小板植入时间为11（8～28）d。8例患者获得长期稳定植入，3例患者植入失败，其中2例行二次移植（1例获得到长期稳定植入，另1例造血重建后再次发生植入失败）。中位随访时间为53（5～135）个月，全部11例患者均存活，其中9例患者外周血象长期正常。

**结论:**

syn-HSCT治疗AA可获得良好的长期生存，植入失败仍是有待解决的主要问题。

## Abstract (en)

**Objective:**

To evaluate the efficacy of syngeneic hematopoietic stem cell transplantation in the treatment of aplastic anemia.

**Methods:**

The clinic data of 11 patients with aplastic anemia undergoing syngeneic HSCT were retrospectively analyzed.

**Results:**

Among all of the 11 patients with AA, 4 males and 7 females were determined, with a median age of 22（7–44）years old. All of the 11 patients achieved engraftment after the first transplantation: neutrophils engraftment occurred after a median of 10 days（range 8–23）, and platelet engraftment occurred after a median of 11 days（range 8–28）. Eight patients achieved long-term stable engraftment: three patients had graft failure, and two of them underwent secondary transplantation （1 case achieved long-term stable engraftment, but graft failure occurred again after hematopoietic reconstruction in another case）. The median follow-up time was 53（5–135）months. All of the 11 patients survived, and the blood routine of 9 patients was normal for a long time.

**Conclusion:**

Syngeneic hematopoietic stem cell transplantation has a good long-term survival rate in the treatment of aplastic anemia, and graft failure is still the most significant problem.

再生障碍性贫血（AA）是一种由机体异常免疫应答介导的骨髓衰竭性疾病[1]，重型再生障碍性贫血（SAA）严重危及患者生命，目前的主要治疗方法为免疫抑制治疗（IST）和异基因造血干细胞移植（allo-HSCT）[2]–[3]。国外研究数据表明，同基因造血干细胞移植（syn-HSCT）能够获得较高的生存率和较低的移植相关死亡率[4]–[5]，国内目前仅有个案报道[6]–[10]。本研究回顾性分析了2009年9月至2020年7月在我院造血干细胞移植中心接受syn-HSCT的11例AA患者的临床资料，结果报告如下。

## 病例与方法

1. 患者及供者资料：我中心于2009年9月至2020年7月收治的11例AA患者，参照《血液病诊断及疗效标准（第四版）》[11]明确诊断并排除先天性骨髓衰竭。男4例，女7例，中位年龄为22（7～44）岁，移植前中位病程为1（1～216）个月。包括SAA-Ⅰ 8例，SAA-Ⅱ 1例，慢性再生障碍性贫血（CAA） 1例，肝炎相关再生障碍性贫血（HAAA）1例。SAA患者中极重型再生障碍性贫血（VSAA）3例。全部11例患者中，8例移植前曾给予粒细胞集落刺激因子（G-CSF）促造血治疗，7例接受过以口服环孢素A （CsA）为主的免疫抑制治疗，5例患者曾应用雄激素类药物治疗，3例患者曾应用左旋咪唑治疗，所有患者均未接受过抗胸腺细胞球蛋白（ATG）治疗。3例患者移植前检出粒细胞PNH克隆，所有患者均未检出红细胞PNH克隆。移植前复查骨髓象：3例患者骨髓细胞增生程度为“增生活跃（−）”至“增生活跃”，8例为“增生重度减低”至“增生减低”。3例患者移植前应用二代测序技术进行了骨髓标本基因突变监测，具体结果见表1。11例患者移植前中位红细胞输注量为24（6～86）U，中位血小板输注量为240（128～720）U。其中2例患者合并2型糖尿病，1例患者移植前发生口腔真菌感染，经抗真菌治疗好转，2例患者移植前合并侵袭性真菌病（IFD），均为肺感染（其中1例抗真菌治疗时间超过1个月，在感染无明显好转的状态下进行移植）。全部患者均具有经STR检测证实的同卵双生的双胞胎供者。患者详细资料见表1。

**表1:** 11例接受同基因造血干细胞移植再生障碍性贫血患者的一般资料

例号	性别	年龄（岁）	诊断	移植前病程（月）	移植前治疗	移植前PNH克隆	移植前骨髓增生程度	移植前红细胞输注量（U）	移植前血小板输注量（U）	移植前骨髓基因突变
1	女	7	SAA-Ⅰ	1	无	无	增生重度减低	6	128	未检测
2	男	22	SAA-Ⅱ	216	司坦唑醇	无	增生活跃	62	144	未检测
3	女	26	SAA-Ⅰ	12	CsA、达那唑、司坦唑醇、左旋咪唑、G-CSF	粒细胞PNH克隆9.39%	增生活跃（−）	32	272	未检测
4	男	12	SAA-Ⅰ	1	CsA、左旋咪唑、达那唑、G-CSF	粒细胞PNH克隆6.61%	增生重度减低	24	240	未检测
5	女	44	SAA-Ⅰ（VSAA）	1	CsA、G-CSF	无	增生重度减低	12	224	未检测
6	女	25	HAAA	1	G-CSF	无	增生减低	34	400	未检测
7	男	15	SAA-Ⅰ	1	CsA、G-CSF	无	增生减低	24	272	未检测
8	女	29	SAA-Ⅰ（VSAA）	1	CsA、左旋咪唑、司坦唑醇	无	增生减低	22	224	未检测
9	女	28	SAA-Ⅰ	1	CsA、rhTPO、rhEPO、G-CSF	粒细胞PNH克隆2.6%	增生减低	24	432	ARID1A基因p.S1948N突变，突变频率48.9%；IDH1基因p.Y208C突变，突变频率46.2%
10	女	20	CAA	73	CsA、达那唑、G-CSF	无	增生活跃	86	720	EP300基因p.S507G突变，突变频率49.4%；NOTCH1基因p.E848K突变，突变频率48.9%
11	男	17	SAA-Ⅰ（VSAA）	2	G-CSF	无	增生重度减低	16	192	KMT2C基因p.V375I突变，突变频率5.9%；TERT基因p.I587T突变，突变频率49.3%；PRK-DC基因p.I1576V突变，突变频率48.4%

注：SAA-Ⅰ：重型再生障碍性贫血Ⅰ型；SAA-Ⅱ：重型再生障碍性贫血Ⅱ型；VSAA：极重型再生障碍性贫血；CAA：慢性再生障碍性贫血；CsA：环孢素A；G-CSF：粒细胞集落刺激因子；rhTPO：重组人血小板生成素；rhEPO：重组人红细胞生成素；PNH：阵发性睡眠性血红蛋白尿症

2. 预处理方案：9例患者采用猪抗人胸腺细胞球蛋白（pATG）（武汉生物制品研究所产品）20 mg·kg^−1^·d^−1^×5 d，氟达拉滨（Flu）30 mg·m^−2^·d^−1^×5 d，环磷酰胺（Cy）总量120～150 mg/kg（分3～4 d连续给药）。其中１例患者加用白消胺（Bu）3.2 mg·kg^−1^·d^−1^×2 d。1例患者采用兔抗人胸腺细胞球蛋白（rATG）（法国赛诺菲公司产品）2.5 mg·kg^−1^·d^−1^×5 d，Flu 30 mg·m^−2^·d^−1^×5 d，Cy总量150 mg/kg（分3 d连续给药）；1例患者采用rATG 2.0 mg·kg^−1^·d^−1^×4 d，联合Cy 160 mg/kg（分4 d连续给药）。预处理相关不良反应依据WHO制定的化疗急性和亚急性不良反应分级标准[12]判定。

3. 造血干细胞采集与输注：全部11例患者的移植物均为同卵双生双胞胎供者的外周血造血干细胞。采用G-CSF 5～10 µg·kg^−1^·d^−1^进行供者造血干细胞动员，5 d后采集外周血干细胞。采集后的细胞悬液进行单个核细胞（MNC）计数、CD34^+^细胞计数和淋巴细胞亚群检测。中位单个核细胞（MNC）输注量为8.00（5.53～16.72）×10^8^/kg，中位CD34^+^细胞输注量为3.56（1.61～4.40）×10^6^/kg，中位CD3^+^T细胞输注量为12.25（4.67～17.72）×10^7^/kg。

4. 植入失败的预防：10例患者应用CsA预防，其中8例于移植前1 d开始CsA 2mg·kg^−1^·d^−1^静脉滴注，胃肠道功能恢复后改为口服用药，2例于移植前1 d起口服CsA 2 mg·kg^−1^·d^−1^。维持初始血CsA浓度200～400 µg/L，分别于移植后1～25个月停用（例10、例11至随访截止未停CsA）。全部11例患者中仅1例（例2）未应用CsA。

5. 支持治疗：所有患者均住百级层流病房，移植前应用复方磺胺噁唑2 g/d×7 d预防耶氏肺孢子菌肺炎，静脉滴注更昔洛韦10 m·kg^−1^·d^−1^×7 d或膦甲酸钠90 mg·kg^−1^·d^−1^×7 d预防巨细胞病毒感染，移植后给予氟康唑（5例）、泊沙康唑（1例）、伊曲康唑（1例）、伏立康唑（1例）预防真菌感染，1例未应用预防真菌药物。2例患者移植前合并肺IFD，预处理期间应用卡泊芬净治疗，移植后给予伏立康唑静脉输注治疗。

6. 定义：粒细胞植入：患者外周血中性粒细胞绝对计数（ANC）>0.5×10^9^/L持续3 d；血小板植入：血小板计数（PLT）>20×10^9^/L持续7 d且脱离血小板输注。原发性植入失败：移植后28 d时中性粒细胞及血小板计数仍未达到造血重建标准；继发性植入失败：造血重建后再次出现持续ANC<0.5×10^9^/L及PLT<20×10^9^/L。移植后14 d、28 d及3、6、9、12、24、36、60个月进行血常规、骨髓细胞形态学及骨髓造血祖细胞培养检测。

7. 随访：随访截至2020年12月1日，病例资料来自住院/门诊病历和电话随访记录。

## 结果

1. 预处理相关不良反应：11例患者中1例出现恶心呕吐等胃肠道反应，1例出现Ⅰ度口腔溃疡，4例出现Ⅰ度肝功能异常，1例出现Ⅲ度肝功能异常（表现为转氨酶升高）；4例患者发生Ⅰ度心功能损害，表现为窦性心动过速，B型钠尿肽前体增高；3例患者出现皮疹，其中1例合并反复发热，考虑血清病反应，应用糖皮质激素治疗后好转。随访期间未观察到预处理相关肺、中枢神经系统及外周神经系统毒性。

2. 移植结果：全部11例患者中位粒细胞植入时间为10（8～23）d，中位血小板植入时间为11（8～28）d。其中8例患者在首次移植后获得长期稳定植入，3例患者发生继发性植入失败（例2、例3、例10）。详见表2。

**表2:** 11例接受同基因造血干细胞移植再生障碍性贫血患者的移植结果

例号	预处理方案	MNC输注量（×10^8^/kg）	CD34^+^细胞输注量（×10^6^/kg）	CD3^+^T细胞输注量（×10^7^/kg）	粒细胞植入（d）	血小板植入（d）	应用CsA	移植后CsA减停时间（月）	随访时间（月）	随访结果
1	rATG+Cy+Flu	7.90	3.56	12.25	23	12	是	1	135	存活
2	rATG+Cy	5.53	2.49	4.84	10	9	否	／	129	存活（输血依赖）
3	pATG+Cy+Flu	7.00	2.45	4.76	9	8	是	3	89	存活（二次移植后血象稳定）
4	pATG+Cy+Flu	8.00	3.04	15.32	13	14	是	25	79	存活
5	pATG+Cy+Flu	10.00	2.00	8.82	10	14	是	13	72	存活
6	pATG+Cy+Flu	6.65	4.32	16.91	10	10	是	14	53	存活
7	pATG+Cy+Flu	7.00	2.38	17.72	11	28	是	15	49	存活
8	pATG+Cy+Flu	6.00	3.72	13.33	8	8	是	16	37	存活
9	pATG+Cy+Flu	16.72	1.61	10.08	8	11	是	12	29	存活
10	Bu+pATG+Cy+Flu	8.00	4.40	7.18	13	20	是	未减停	20	存活（二次移植后需口服药物）
11	pATG+Cy+Flu	12.00	3.72	13.97	10	11	是	逐渐减量	5	存活

注：rATG：兔抗人胸腺细胞球蛋白；pATG：猪抗人胸腺细胞球蛋白；Cy：环磷酰胺；Flu：氟达拉滨；Bu：白消安；MNC：单个核细胞；CsA：环孢素A；／：不适用

3. 并发症：11例患者中，3例预处理期间及化疗后骨髓抑制期发生血流感染，其中2例病原菌为大肠埃希菌，1例病原菌为凝固酶阴性溶血葡萄球菌，经抗细菌治疗后均治愈。1例患者移植后20 d出现肺IFD，为移植前肺IFD病情进展，经过抗真菌治疗后得到控制。所有患者移植后均未发生巨细胞病毒血症。

4. 随访结果：截止2020年12月，中位随访时间为53（5～135）个月。全部11例患者均存活，9例患者长期血象正常（8例已停用CsA口服，1例CsA减量中）。1例植入失败患者需要输血支持治疗，另1例患者仍口服CsA、艾曲泊帕等药物治疗。

## 讨论

国内以及国外的一些研究报道了同基因骨髓移植的个案[13]–[16]。Hinterberger等[5]回顾了1964年至1992年进行同基因骨髓移植的40例AA患者，其中23例患者在没有接受预处理的情况下接受移植，其中仅有7例血液学完全恢复，植入失败的患者分别接受了2～5次包含预处理的移植，其中13例患者骨髓完全恢复。首次移植前给予预处理的17例患者中，12例顺利植入。首次移植前进行预处理的患者血液学恢复的可能性更大，但这部分患者移植相关死亡率相对更高。但有部分病例，虽然移植前使用了环磷酰胺/全身放疗预处理，也未能引发持续稳定的血液学恢复[17]，提示AA还存在着另一种与骨髓微环境异常相关的发病机制。

Gerull等[4]对欧洲血液和骨髓移植学会（EBMT）1976年至2009年间接受syn-HSCT的88例AA患者进行了回顾性分析，10年总生存率为93％，有5例患者发生移植相关死亡，植入失败发生率为32％。在近20年的研究中，包含免疫抑制预处理以及应用外周血干细胞的移植得到了越来越多的应用，研究发现，外周血干细胞移植比骨髓移植造血重建更快，并且植入率更高，而未应用预处理的患者植入失败的风险更大，缺乏移植后的免疫抑制也可能会造成移植失败的风险增加。根据以上结果得出syn-HSCT前必须进行预处理，清除患者体内存在的异常免疫环境，同时移植后要给予免疫抑制剂，才能保证提高疗效。

本组接受syn-HSCT的11例患者均获得长期生存，首次移植后均获得快速造血重建，3例患者发生继发性植入失败，低于文献[4]报道的植入失败率（32％），这可能与我们应用外周血干细胞作为移植物并在移植前给予预处理有关。3例植入失败患者病史均较长（移植前病程分别为216、12、73个月），移植前多部位骨髓涂片增生程度为活跃（−）至活跃，而其他8例获得长期稳定植入患者移植前骨髓增生程度为重度减低～减低。例2在移植后未给予CsA作为免疫抑制治疗，这与国外研究中不应用移植后免疫抑制剂的患者植入失败率较高的趋势相符合。

以往研究显示，allo-HSCT患者植入失败的发生与预处理方案相关[18]–[19]。我中心以往采用减低剂量Cy联合ATG及Flu进行allo-HSCT前预处理（rATG 2.5 mg·kg^−1^·d^−1^或pATG 20 mg·kg^−1^·d^−1^连续5 d，Flu 30 mg·m^−2^·d^−1^，连续5 d，Cy总量120～150 mg·kg^−1^·d^−1^分连续3～4 d应用），获得了较高的植入率[20]–[21]。近年来国外的一些syn-HSCT报道中，为了达到持久的血液学恢复，给予较大剂量ATG联合Cy或者Flu联合TBI预处理以加强免疫抑制，取得了较好的植入效果[22]–[25]。我们认为，AA移植预处理多数为非清髓，移植后患者体内仍残存异常免疫细胞。syn-HSCT因为免疫原性非常接近，不能通过移植物抗宿主反应清除患者体内残留的异常免疫细胞。需要通过加强预处理的免疫抑制强度来达到清除异常免疫细胞的目的。本研究中，10例患者采用较强的ATG+Cy+Flu预处理方案，获得稳定植入。发生植入失败的例2患者采用的预处理方案是免疫抑制强度较弱的ATG+Cy方案，提示适当的增加syn-HSCT预处理免疫抑制强度，可能有利于达到稳定植入；对于病程较长，骨髓增生尚可的患者更需要加强预处理强度。另外，我们认为移植后免疫抑制剂CsA的应用可以提高植入率，同时应该注意CsA应用的剂量与疗程。

以往allo-HSCT的研究结果显示，回输有核细胞以及CD34^+^细胞较少的患者[18]–[19]，更易发生植入失败；并且，由于供者细胞毒性T细胞以及CD4^+^ CD25^+^Foxp3^+^调节T细胞（Treg）均有利于植入，去除T细胞的allo-HSCT植入失败率更高[19]。本研究发生植入失败的3例患者，CD3^+^T细胞输注量均少于其他患者。例3二次移植输注的MNC及CD3^+^T细胞数均明显高于首次移植，最终获得长期稳定植入。因此我们推测在syn-HSCT中，CD3^+^T细胞输注量可能与植入率相关。

国外研究显示，首次经预处理的移植失败后给予二次移植，有三分之二的患者可获得稳定植入，仍有少数患者再次发生植入失败[4]–[5]。本组病例中，例2因为个人原因未行二次移植；例3二次移植后血象迅速恢复并达到长期稳定植入；例10二次移植后有短暂的血液学恢复，仍出现血象下降，需服用促造血药物，目前血象未完全恢复。我们推测反复移植失败可能与AA发病的其他机制相关。例11的骨髓中检测到TERT基因p.I587T突变，此突变见于先天性角化不良常染色体显性遗传4型中，编码端粒酶相关成分[26]，但其供者的二代测序未发现该突变，我们认为，同卵双胞胎出现突变与否，可能与AA发病有关，但这些突变是否会对植入失败有潜在影响尚需继续研究。

syn-HSCT是AA患者宝贵的治疗机会，具有良好的长期生存率和较低的移植相关死亡率。本组病例结果显示，syn-HSCT治疗AA可获得良好的长期生存，植入失败仍是主要问题，移植方案尚需进一步优化。

## References

[1] Young NS (2018). Aplastic Anemia[J]. N Engl J Med.

[2] Iftikhar R, Chaudhry QUN, Anwer F (2021). Allogeneic hematopoietic stem cell transplantation in aplastic anemia: current indications and transplant strategies[J]. Blood Rev.

[3] Aljurf M, Al-Zahrani H, Van Lint MT (2013). Standard treatment of acquired SAA in adult patients 18-40 years old with an HLA-identical sibling donor[J]. Bone Marrow Transplant.

[4] Gerull S, Stern M, Apperley J (2013). Syngeneic transplantation in aplastic anemia: pre-transplant conditioning and peripheral blood are associated with improved engraftment: an observational study on behalf of the Severe Aplastic Anemia and Pediatric Diseases Working Parties of the European Group for Blood and Marrow Transplantation[J]. Haematologica.

[5] Hinterberger W, Rowlings PA, Hinterberger-Fischer M (1997). Results of transplanting bone marrow from genetically identical twins into patients with aplastic anemia[J]. Ann Intern Med.

[6] 马 凤宇, 冯 慧敏, 高 峰 (2020). 不含预处理的同基因外周血造血干细胞移植治疗重型再生障碍性贫血获得长期缓解一例[J]. 中华内科杂志.

[7] 袁 成录, 王 玲, 史 春雷 (2006). 同基因外周血造血干细胞移植治疗再生障碍性贫血[J]. 齐鲁医学杂志.

[8] 王 蕊, 温 丙昭, 江 明 (2005). 二次同基因外周血干细胞移植治疗重型再生障碍性贫血Ⅰ型1例[J]. 临床血液学杂志.

[9] 杨 光, 梁 红, 郝 文鹏 (2005). 同基因外周血造血干细胞移植成功治疗肝炎后重型再生障碍性贫血1例报告[J]. 临床血液学杂志.

[10] 李 颖, 袁 成录, 史 春雷 (2006). 同基因外周血造血干细胞移植治疗慢性再生障碍性贫血一例[J]. 中华血液学杂志.

[11] 沈 娣, 赵 永强 (2018). 血液病诊断及治疗标准[M].

[12] National Cancer Institute (1998). Common toxicity criteria: index [S/OL].

[13] Lu DP (1981). Syngeneic bone marrow transplantation for treatment of aplastic anaemia: report of a case and review of the literature[J]. Exp Hematol.

[14] Champlin RE, Feig SA, Sparkes RS (1984). Bone marrow transplantation from identical twins in the treatment of aplastic anaemia: implication for the pathogenesis of the disease[J]. Br J Haematol.

[15] Niki T, Nakao S, Ueda M (1990). Incomplete marrow recovery associated with hepatitis after syngeneic bone marrow transplantation for aplastic anaemia: successful treatment with second marrow transplantation without preconditioning[J]. Br J Haematol.

[16] Appelbaum FR, Cheever MA, Fefer A (1985). Recurrence of aplastic anemia following cyclophosphamide and syngeneic bone marrow transplantation: evidence for two mechanisms of graft failure[J]. Blood.

[17] Appelbaum FR, Fefer A, Cheever MA (1980). Treatment of aplastic anemia by bone marrow transplantation in identical twins[J]. Blood.

[18] Mattsson J, Ringdén O, Storb R (2008). Graft failure after allogeneic hematopoietic cell transplantation[J]. Biol Blood Marrow Transplant.

[19] Olsson R, Remberger M, Schaffer M (2013). Graft failure in the modern era of allogeneic hematopoietic SCT[J]. Bone Marrow Transplant.

[20] 陈 欣, 魏 嘉磷, 黄 勇 (2014). 异基因造血干细胞移植治疗重型再生障碍性贫血70例的疗效分析[J]. 中华器官移植杂志.

[21] 黄 勇, 何 祎, 刘 欣 (2017). 减低剂量环磷酰胺的预处理方案对重型再生障碍性贫血同胞全相合外周血造血干细胞移植疗效的影响[J]. 中华血液学杂志.

[22] Hwang WL, Yang Y, Chen GR (2000). Syngeneic peripheral blood stem cell transplantation with brief immunosuppression for severe aplastic anemia[J]. Bone Marrow Transplant.

[23] Savic A, Balint B, Urosevic I (2010). Syngeneic peripheral blood stem cell transplantation with immunosuppression for hepatitis-associated severe aplastic anemia[J]. Turk J Haematol.

[24] El-Cheikh J, Atoui A, Moukalled N (2019). Successful treatment of severe aplastic anemia with syngeneic stem cell transplantation in the setting of active disseminated mucormycosis[J]. Med Mycol Case Rep.

[25] Kook H, Kim GM, Kim HJ (2000). Rubella-associated aplastic anemia treated by syngeneic stem cell transplantations[J]. Am J Hematol.

[26] Yamaguchi H, Calado RT, Ly H (2005). Mutations in TERT, the gene for telomerase reverse transcriptase, in aplastic anemia[J]. N Engl J Med.

